# Exenatide Is Neuroprotective in a New Rabbit Model of Hypoxia-Ischemia

**DOI:** 10.3390/cells14211715

**Published:** 2025-11-01

**Authors:** Eridan Rocha-Ferreira, Malin Carlsson, Pernilla Svedin, Kerstin Ebefors, Owen Herrock, Anna-Lena Leverin, Henrik Hagberg

**Affiliations:** 1 Institute of Clinical Sciences, Sahlgrenska Academy, University of Gothenburg, 405 30 Gothenburg, Sweden; carlssonmalin99@gmail.com (M.C.); owen.herrock@gu.se (O.H.); anna-lena.leverin@neuro.gu.se (A.-L.L.); henrik.hagberg@obgyn.gu.se (H.H.); 2 Institute of Neuroscience and Physiology, Sahlgrenska Academy, University of Gothenburg, 405 30 Gothenburg, Sweden; pernilla.svedin@fysiologi.gu.se (P.S.);

**Keywords:** hypoxia-ischemia, exenatide, rabbit model, brain injury, neonatal

## Abstract

Hypoxia-ischemia is a serious perinatal complication affecting neonates globally. Animal models have increased the understanding of its pathophysiology and have been used to investigate potential therapies. Exenatide, clinically used for the treatment of type 2 diabetes mellitus, also protects the rodent brain from hypoxia-ischemia. The rabbit brain has an earlier neurodevelopmental maturation than rodents, as well as similar postnatal maturation to humans. We hereby introduce a new, reproducible hypoxia-ischemia model in rabbit kits at postnatal day (P) 3–4. Following hypoxia-ischemia, rabbit kits received different exenatide concentrations: 170 μg/g (2-dose) or 500 μg/g (1- or 2-dose), or vehicle. The brains were collected seven days later for histological assessment showing that 500 μg/g exenatide, either as a 1- or 2-dose regimen, reduced brain tissue loss by 90% in hypoxia-ischemia experiments both at P3 and P4. A second cohort received a 1-dose 500 μg/g of exenatide or vehicle, and were sacrificed at different early time-points for glucose, ketone bodies, body weight, and temperature measurements. Our results showed a transient 2-fold increase in ketone bodies (0.6 to 1.3 mmol/L) at 6 h. Exenatide did not affect glucose, body temperature or weight gain and appears to be safe and well tolerated in the rabbit model of hypoxia-ischemia.

## 1. Introduction

Neonatal hypoxia-ischemia is a major cause of brain damage in newborns. It occurs around the time of birth and can result in mild to severe neurodisabilities and even death. In survivors, there is a high risk of motor disability such as cerebral palsy as well as other neurological impairments, including epilepsy [1].

Animal models of hypoxia-ischemia have helped to shed light on its pathophysiological progression and to evaluate the efficacy of novel therapeutics. However, humans, unlike many other mammals, develop their brain perinatally [2]. Therefore, it is important to use animal species that better mimic human brain development when considering a model of birth asphyxia. In rodents, their lissencephalic brain development occurs postnatally. Additionally, cerebral blood flow, and gray and white matter ratios are substantially different in rodents compared to in humans, which is likely to be associated with differences in age-dependent brain regions’ vulnerability to injury [3]. Despite producing similar anatomical injuries as in human pediatric disorders, motor function deficits are harder to establish using rodent models [4]. Meanwhile, large animal models like sheep and nonhuman primates have a prenatal brain development, with near complete myelination of the brain and advanced motor development compared to humans by the time of birth. Additionally, ethical and economic considerations cause large-scale drug screening to be implausible in these species, leaving researchers with a few options of relevant animal models.

Although newborn rabbits are considered to have the brain maturation equivalent to 26–34 weeks human gestation [5], rabbits have perinatal brain development. This includes limited neurobehavior at birth and the postnatal start of myelination, similarly to humans [6,7]. Additionally, rabbits have several brain similarities to humans occurring during the neonatal period, including microglial presence within the white matter tracts and oligodendrocyte maturation, with parallel brain growth when compared to humans [8,9,10]. In rabbits, peak myelination occurs at postnatal day 5 (P5), with developed cortical layers, despite reduced visual and auditory development [11,12,13]. Similarly to humans, rabbits before P5 have limited mobility/autonomy, as indicated in a report by Zhang and colleagues who found that after P5–P7, rabbit kits achieve further developmental milestones, such as righting reflex and cliff avoidances, but not yet eye opening, head and body elevation, and hopping [4].

Previous rabbit models of neonatal brain injury have resulted in preterm injury and used either maternal aortic occlusion [14] or occlusion of both uterine arteries [15]. The only publication on a postnatal birth asphyxia model that we found was performed by D’Arceuil and colleagues, who at P8–P10 used a combination of ligation of the common carotid artery and exposure to hypoxia (10%, up to 4 h). However, their studies focused mostly on MRI, with limited assessments of histopathological consequences [5,16,17,18]. Therefore, our first study aim was to induce hypoxia-ischemia in the rabbits at P3–P5, to better mimic the newborn human brain at term. Following this, the second aim of our study was to use this newly established rabbit model of hypoxia-ischemia to investigate whether exenatide, a glucagon-like peptide (GLP)-1 receptor agonist clinically used as treatment of type 2 diabetes mellitus, could attenuate brain injury in neonatal rabbits.

## 2. Materials and Methods

### 2.1. Animals

All experiments conformed to the Swedish Board of Agriculture and were approved by the Gothenburg Animal Ethics Committee (3256/20 and 4309/22) for the use of New Zealand white rabbits (Envigo, London, UK). All animals had free access to chow and water, received food enrichment pellets daily, and were maintained at a 12 h light/dark cycle. Breeding of dams was performed in-house, and adult females were only put into breeding every 4–6 months. Kits of both sexes were used for all experiments and the ARRIVE guidelines were followed throughout the study.

### 2.2. Surgery

**Figure 1:**
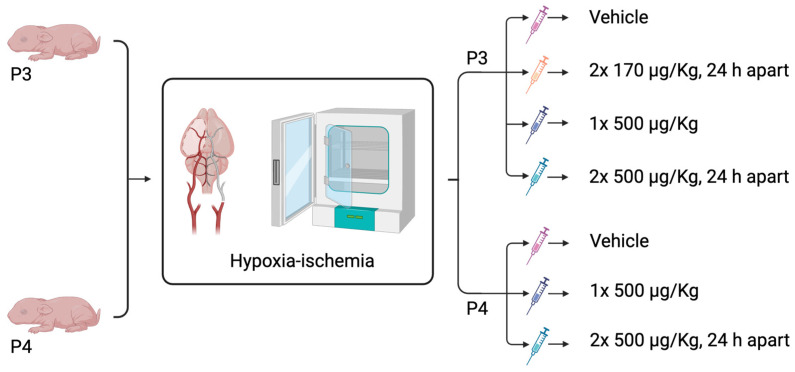
Schematic diagram of ExAc different dosing treatment regimens following hypoxia-ischemia either at P3 or P4. Created in BioRender. Rocha Ferreira, E. (2025) https://BioRender.com/i9yz99f (accessed on 19 August 2025).

To better represent human newborn brain maturation, rabbit kits underwent hypoxia-ischemia at P3–P5. Kits were anesthetized with isoflurane (5% induction and 1.5% maintenance) and the left common carotid artery was permanently ligated with suture. After 1 h recovery in a warmed plate (35 °C), kits were placed in a hypoxic chamber (6–8% O_2_, 36 °C) for 90 min (P3–P5), 150 min, 210 min, or 250 min (P3–P4) (Figure 1).

### 2.3. Exenatide Acetate (ExAc) Administration

Good manufacturing practice (GMP)-certified exenatide acetate (ExAc, 4019602.1000, Bachem, Bubendorf, Switzerland) was administered using three different intraperitoneal (i.p.) dosing regimens in this study. All dosing strategies began within 10 min of the 210 min HI exposure. Rabbit kits were randomized to either vehicle, two doses of 170 μg/kg ExAc 24 h apart, one dose of ExAc 500 μg/kg, or two doses of 500 μg/kg ExAc 24 h apart. HyPure Cell Culture Grade Water, endotoxin free (SH30529.01, Cytiva, Gothenburg, Sweden; 0.5 μg/g body weight), was used to dissolve the exenatide acetate; therefore, it was given as a vehicle control to untreated kits. Body weight was measured at start of hypoxia-ischemia and at each injection time-point (Figure 1).

### 2.4. Measurements of Glucose, Ketone Bodies, and Temperature

A cohort of rabbits underwent hypoxia-ischemia at P4, consisting of 6% O_2_ and 210 min hypoxia, followed by a single dose of ExAc (500 μm/kg) or ultrapure water (vehicle, 5 μg/g body weight). At 45 min, 6 h, or 7 d animals were terminated, and blood glucose (WELL10-15) and ketone bodies (WELL10-10KET) levels (mmol/L) were measured using a Wellion GALILEO meter (Wellion, Malmö, Sweden). Core temperature was measured using a rectal probe (T21, 0.41 mm diameter) and body weight was also recorded at 45 min, 6 h, and 7 d.

### 2.5. Histology Preparation

Seven days after hypoxia-ischemia, a time-point when the secondary phase of brain injury was completed [19], kits were terminated, and the brains collected and stored in Histofix (Histolab, Askim, Sweden) for 7 d before being dehydrated and embedded in paraffin blocks. Additionally, naïve P3, P4, and P5 brains were also collected in the same manner. Using a microtome, 7 μm thick coronal brain sections were produced and collected based on the rabbit brain anatomy [20], and were collected on superfrost plus slides at six different levels across the striatum and hippocampus brain regions.

### 2.6. Immunohistochemistry

Slides were prepared for immunohistochemical staining by deparaffination in xylene, followed by rehydration in graded alcohol. Sections were then boiled in 0.01 M citric acid buffer (pH 6.0) for antigen recovery, and blocked for endogenous peroxidase (3% H_2_O_2_) and nonspecific binding with horse serum. The sections were then incubated with mouse anti-microtubule-associated protein-2 (MAP-2, M4402, Merck, Solna, Sweden) at 4 °C overnight followed by 1 h incubation with biotinylated horse anti-mouse antibody, (1:250, BA2001, Vector Laboratories, Solna, Sweden). After incubation with Avidin-Biotin enzyme Complex (ABC-Elite, Vector Laboratories), the immunoreactivity was visualized using 3.3-di-aminobenzidine—DAB (0.5 mg/mL) enhanced with nickel sulfate (15 mg/mL). All sections were dehydrated in increasing concentrations of alcohol and xylene, and were covered with a coverslip using Pertex (00840-05, Histolab, Askim, Sweden).

### 2.7. Immunofluorescence

Double labeling sections from naïve P4 rabbit kits were deparaffinized, rehydrated, and boiled in citric acid buffer (0.01 M, pH 6.0, 10 min). After blocking in 5% donkey serum for 1 h at room temperature the sections were incubated for 2 h at room temperature for the co-localization of GLP-1R (1:100, NBP-1-97308, Novus Biologicals, Abingdon, UK) with neuronal specific nuclear protein (NeuN, 1:250, MAB377, Millipore, Solna, Sweden), glial fibrillary acidic protein (GFAP, 1:500, G3893, Merck), ionized calcium-binding adapter molecule 1 (Iba1, 1:250, ab5076, Abcam, Cambridge, UK), or oligodendrocyte transcription factor 2 (Olig2, 1:500, MABN50, Millipore). The AlexaFluor secondary antibodies (ThermoFischer, Gothenburg, Sweden), Donkey-anti-Goat 488 (A11055), Donkey-anti-Rabbit 594 (A21207), Goat-anti-mouse 488 (A11001), and Goat-anti-Rabbit 594 (A11012) antibodies were used in 1:500 dilutions in PBS and incubated for 1 h at room temperature. The sections were mounted with a coverslip using ProLong™ Gold + DAPI (P36935, ThermoFisher). Colocalization was examined and photographed using a Zeiss LSM 800 confocal microscope (Zeiss, Oberkochen, Germany). All images were processed using ZEN blue, version 3.4 (Zeiss, Oberkochen, Germany).

### 2.8. Data Analysis

Brain injury scoring at the level of the hippocampus was performed in MAP-2-stained sections. The scale spanned 0, where no brain damage is present, to 4, where there is substantial tissue loss and brain infarction [21].

Brain sections were scanned using Zeiss Axioscan 7 (Zeiss, Oberkochen, Germany) for the quantification of gray matter area loss using MAP-2 staining. A total of six sections were measured, three at the striatum level and three at the hippocampus level. The total area of positive MAP-2 staining in each intact hemisphere in these sections was outlined and measured using ImageJ (version 1.53t, NIH). The percentage of tissue loss was calculated by subtracting the ipsilateral positive area from the corresponding contralateral region. This assessment assumes that the contralateral brain hemisphere equals to 100% of the intact area. However, there are sections where, due to potential natural asymmetry between contralateral and ipsilateral hemispheres, the undamaged ipsilateral hemisphere might be slightly larger, resulting in minimal artefactual negative tissue loss.

### 2.9. Statistical Analysis

All assessments were performed by a blinded researcher, and the resulting data were analyzed using GraphPad Prism 10.0 (GraphPad Software, Boston, MA, USA). The data was first checked for Gaussian distribution using the D’Agostino and Pearson normality test. The Mann–Whitney, Kruskal–Wallis, Dunn’s test or a one-way ANOVA with Tukey’s multiple comparisons test was used when appropriate to determine statistical significance. The test used for each experiment is stated in the figure legends, and *p*-values of <0.05 were statistically significant. The data is expressed as individual animals or mean ± SEM.

## 3. Results

### 3.1. Hypoxia-Ischemia Titration

**Figure 2:**
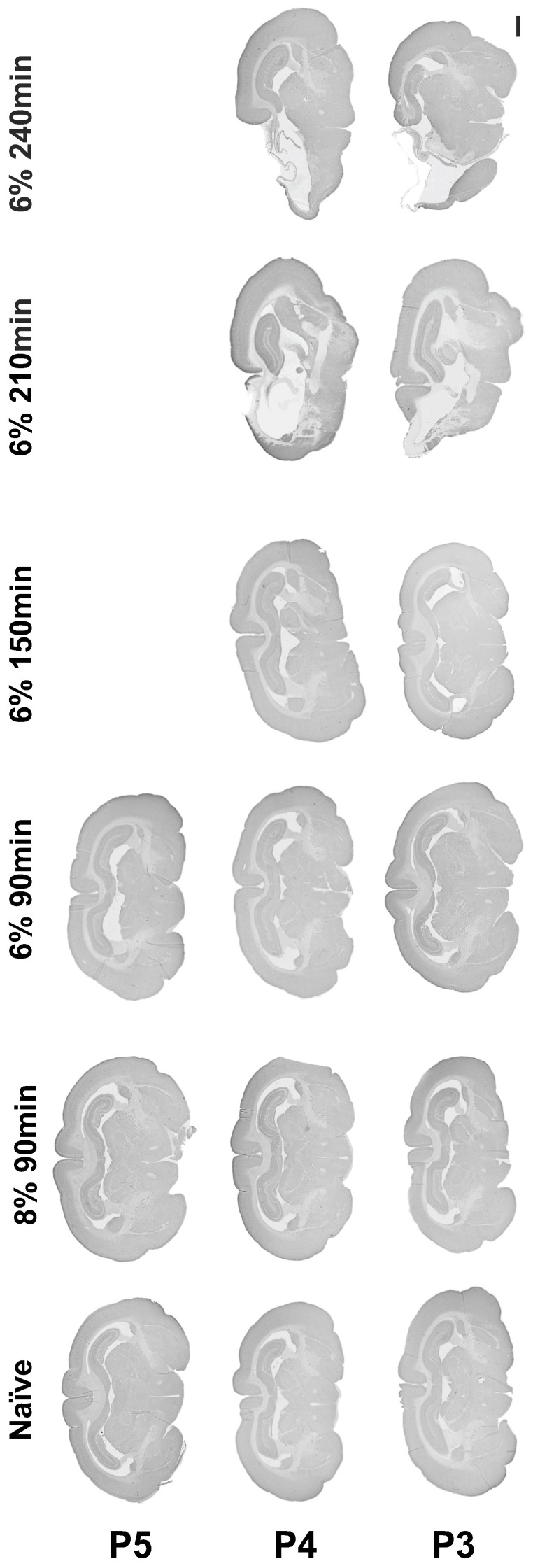
Establishing a rabbit model of hypoxia-ischemia. Micrograph representation of brain injury following hypoxia-ischemia performed between P3 and P5, with gradual reduction in oxygen from 8 to 6% and gradual increase in hypoxia duration from 90 min to 240 min. Scale bar = 2 mm.

Following permanent ligation of the left common carotid artery, oxygen reduction to 8% or 6% for a duration of 90 min did not result in hypoxic–ischemic brain injury at P3, P4, or P5. Oxygen reduction to 6% in both the P3 and P4 animals, with increasing hypoxia duration gradually up to 240 min, resulted in ipsilateral brain injury after exposure for 210 min and 240 min to 6% hypoxia (Figure 2).

### 3.2. GLP-1R Expression

**Figure 3:**
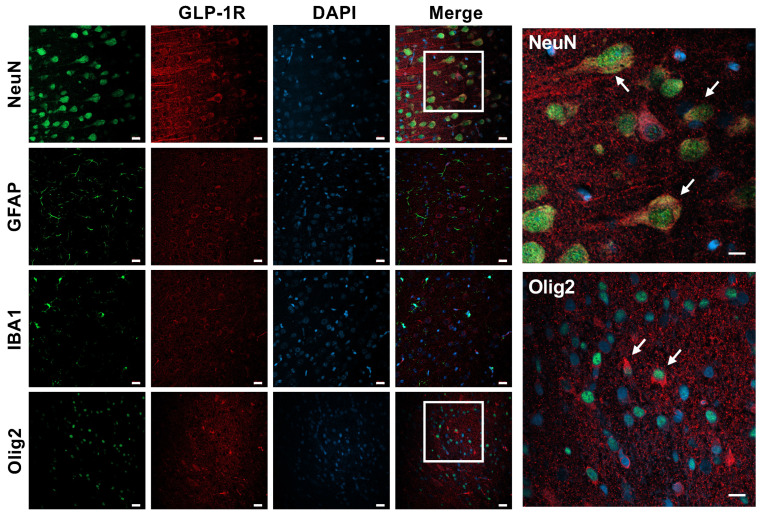
GLP-1R expression in the neonatal rabbit brain. Confocal microscopy imaging demonstrating GLP-1R expression in neurons (NeuN) and to a smaller extent also in oligodendrocytes (Olig2) in the naïve brains of P4 rabbit kits. No colocalization was observed with astroglia (GFAP) or microglia (IBA1) cells. White arrows represent merged cells. Scale bar = 20 μm and = 40 μm (inserts).

Immunofluorescence colocalization with different neural cells showed that the GLP-1R is present in neurons and to a lesser extent in oligodendrocytes, but not in astroglia or microglia (Figure 3).

### 3.3. ExAc Treatment Is Neuroprotective Following Hypoxia-Ischemia at P3

**Figure 4:**
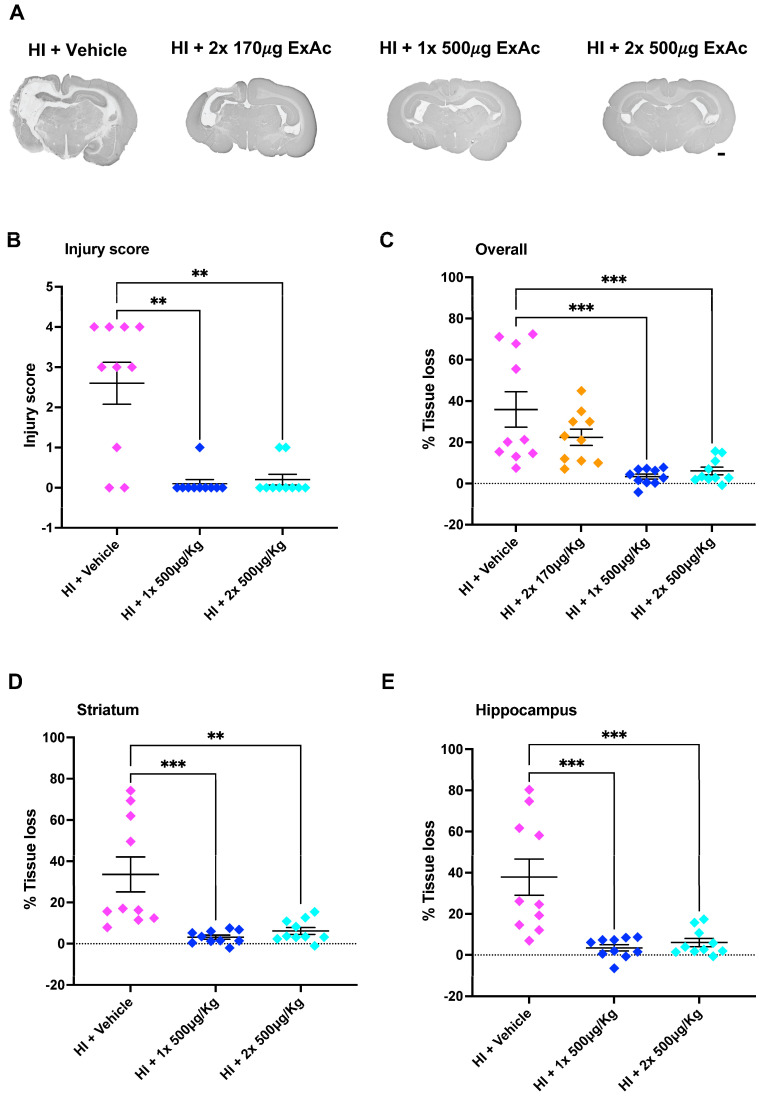
ExAc is neuroprotective following hypoxia-ischemia at P3. (**A**) Whole brain representative micrographs of hypoxia-ischemia plus vehicle control (*n* = 10) at two administrations of 170 μg/kg 24 h apart (*n* = 10), a single administration of 500 μg/kg (*n* = 10) or a double administration of 500 μg/kg 24 h apart (*n* = 10). (**B**) Macroscopic injury score. (**C**) Overall (**D**) striatum, and (**E**) hippocampus level tissue loss measurements. Data presented as individual animals or mean ± SEM and analyzed using Kruskal-Wallis, Dunn’s test (injury score), or one-way ANOVA followed by Tukey’s multiple comparisons test. ** *p* < 0.01 and *** *p* < 0.001. Scale bar = 2 mm.

The initial scoring of brain injury at the level of the hippocampus at 7 d after hypoxia-ischemia showed that ExAc 500 μg/kg, given either as a 1-dose (*p* = 0.0013) or 2-dose (*p* = 0.0045) treatment, reduced brain injury significantly, with minimal injury detected when compared to vehicle-treated controls (Figure 4A,B). Similarly, MAP-2 tissue loss measurements in both 1-dose and 2-dose ExAc 500 μg/kg resulted in an overall brain protection (*p* = 0.0002 and *p* = 0.0006, Figure 4C) that was also significant both at the levels of the striatum (*p* = 0.0006 and *p* = 0.0018, Figure 4D) and hippocampus (*p* = 0.0003 and *p* = 0.0006, Figure 4E).

The attempt at reducing ExAc dose concentration to 170 μg/kg, given as a 2-dose 24 h apart treatment, abolished ExAc-mediated brain protection, and lowering the dose resulted in a significant tissue loss (Figure 4C).

### 3.4. ExAc Treatment Is Neuroprotective Following Hypoxia-Ischemia at P4

Unexpected mortality occurred in some rabbit litters during the hypoxia period, with one in six animals dying in the last 30 min of hypoxia (total duration 210 min). Therefore, the neuroprotective ExAc 500 μg/kg treatment was repeated in a new cohort which underwent hypoxia-ischemia at P4 with 210 min of hypoxia. No mortality occurred at this age.

**Figure 5:**
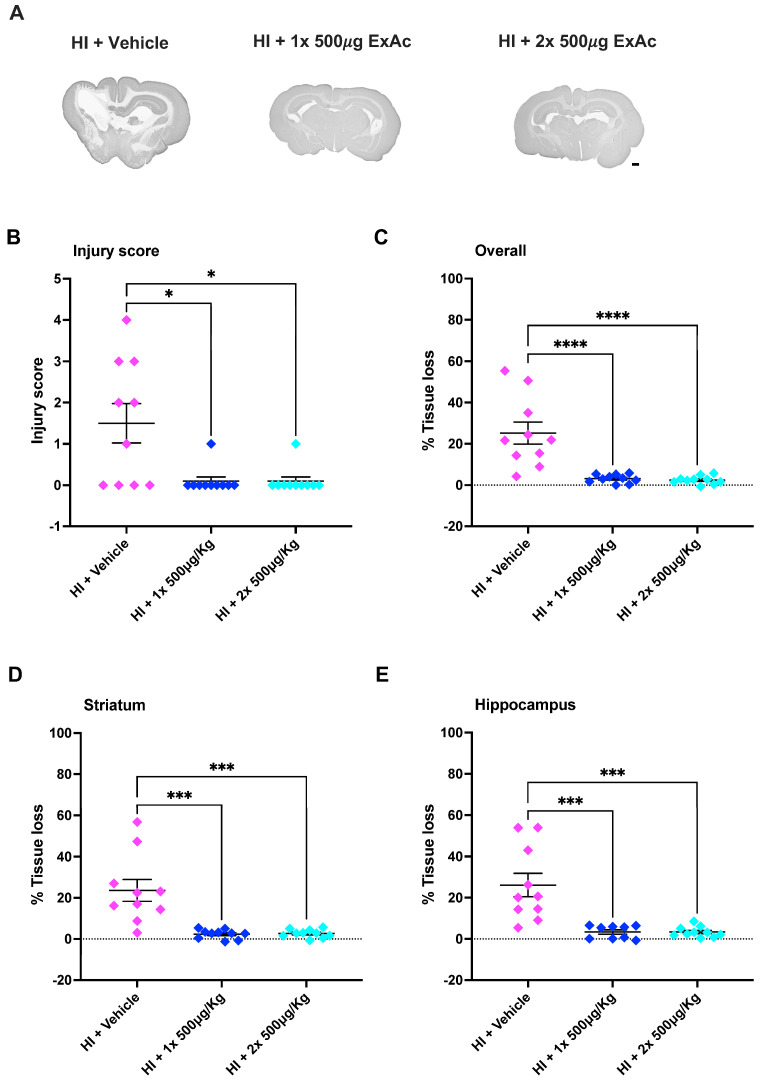
ExAc remains neuroprotective following hypoxia-ischemia at P4. (**A**) Whole brain representative micrographs of hypoxia-ischemia plus vehicle control (*n* = 10) at single administration of 500 μg/kg (*n* = 10) or double administration of 500 μg/kg 24 h apart (*n* = 10). (**B**) Macroscopic injury score. (**C**) Overall (**D**) striatum and (**E**) hippocampus level tissue loss measurements. Data presented as individual animals or mean ± SEM and analyzed using Kruskal-Wallis, Dunn’s test (injury score), or one-way ANOVA followed by Tukey’s multiple comparisons test. * *p* < 0.05, *** *p* < 0.001 and **** *p* < 0.0001. Scale bar = 2 mm.

Brain injury scoring at the level of the hippocampus at 7 d after P4 hypoxia-ischemia showed that treatment with both the 1-dose (*p* = 0.0211) and the 2-dose (*p* = 0.0211) of ExAc 500 μg/kg reduced brain injury substantially compared to the vehicle groups, with minimal brain injury detected in the ExAc groups (Figure 5A,B). Additionally, MAP-2 tissue loss measurements showed that both 1-dose and 2-dose 500 μg/kg ExAc resulted in overall brain protection (*p* < 0.0001 and *p* < 0.0001, Figure 5C). This was also significant at both the striatum (*p* = 0.0001 and *p* = 0.0002, Figure 5D) and hippocampus (*p* = 0.0003 and *p* = 0.0002, Figure 5E) brain levels, when compared to vehicle-injected kits.

### 3.5. Effect of ExAc on Glucose, Ketone Bodies, Body Weight and Temperature

**Figure 6:**
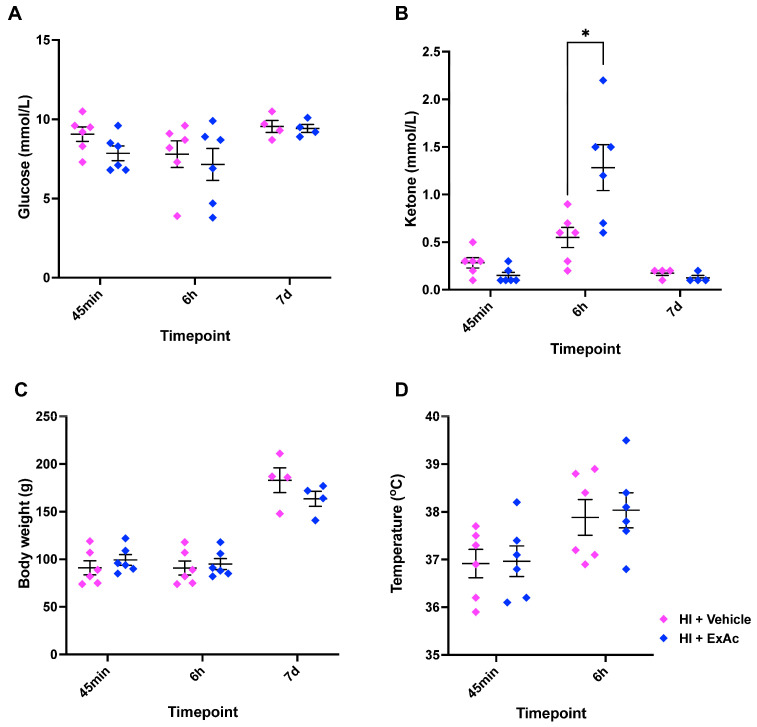
ExAc administration physiological analysis. (**A**) Blood glucose, (**B**) blood ketone bodies, (**C**) body weight, and (**D**) rectal temperature measurements at different time-points. N numbers consisted of hypoxia-ischemia + vehicle (*n* = 6) or 1× 500 μg/kg ExAc (*n* = 6). Data presented as individual animals ± SEM and analyzed using Mann–Whitney test. * *p* < 0.05.

A separate cohort of rabbits underwent hypoxia-ischemia at P4 followed by a single dose of 500 μg/kg ExAc. ExAc-treated animals showed no difference in glucose levels at 45 min, 6 h or 7 d when compared to vehicle-treated animals (Figure 6A). Ketone bodies readout showed a 2-fold increase only at 6 h after hypoxia-ischemia in the ExAc-treated group when compared to the vehicle group (1.3 mmol/L vs. 0.6 mmol/L, *p* = 0.032, Figure 6B). Body weight (Figure 6C) and rectal temperature (Figure 6D) measurements showed no difference between the ExAc and vehicle groups.

## 4. Discussion

Hypoxia-ischemia is a global health burden seriously affecting around 0.75 million babies every year [22]. Despite multiple experimental studies of diverse potential therapeutic agents leading to early-phase clinical trials, therapeutic hypothermia remains the only clinically available treatment for hypoxia-ischemia. However, therapeutic hypothermia is only routinely used in high-income countries, where the hypoxia-ischemia incidence is considerably lower than in low- and mid-income countries [1]. Additionally, following therapeutic hypothermia treatment, up to 29% of treated infants still go on to develop adverse outcomes [23]. This has continued to result in an unmet need to find new therapeutic strategies to ameliorate outcomes in affected infants globally.

In our study, our first aim was to generate a neonatal rabbit model of hypoxia-ischemia, with the aim of bridging the basic and translational models of neonatal hypoxic–ischemic brain injury. Based on our search of the literature, we established that performing hypoxia-ischemia in P3 to P4 rabbit kits most closely corresponds to human newborn brain maturation at term. We successfully created a reproducible model, where continuous exposure to 210 min of 6% oxygen, following the unilateral ligation of the left common carotid artery, resulted in substantial gray matter injury both at the striatum and hippocampal levels, which are brain regions commonly affected in human neonates [1].

We have previously shown that GLP-1Rs are expressed in both the human preterm brain as well as in the neonatal mouse brain. In mice, colocalization of GLP-1Rs occurred mostly in neuronal cells and, to a much lesser extent, in astroglia [24]. In the present study, we first wanted to confirm if GLP-1Rs are present in rabbit kit brains. Our results show that GLP-1Rs are expressed mostly in neurons, but interestingly, also in oligodendrocytes. This is in agreement with previous studies on adult mice that have shown that GLP-1Rs are present in mature oligodendrocytes in multiple sclerosis [25], but ours appears to be the first study showing GLP-1R expression in neonatal oligodendrocytes. These findings are of great relevance because, following neurons, oligodendrocytes are the most vulnerable cells to hypoxia-ischemic injury [26].

Correct dose extrapolation between different species is crucial in developing new pharmaceutical therapies, and it is widely acknowledged that the larger the animal, the lower the dose requirement. Using the data guide from Nair and Jacob [27] based on FDA guidelines, a mouse should require ~12× higher dose than a human, whereas a rabbit would require a dose 3× higher. Therefore, based on our previous mouse studies, where 500 μg/kg was required to achieve substantial neuroprotection, we decided to test, additionally, a dose reduction to 170 μg/kg. As exenatide has a well-known safety profile, with no detectable toxicity in mice, rats, and monkeys, even at chronic administration of doses up to 450× higher than the clinically used dose (0.1–1 μg/kg) (https://www.accessdata.fda.gov/drugsatfda_docs/nda/2009/021919s000pharmr.pdf, accessed on 19 August 2025), our aim was to explore potentially efficacious doses. However, in our current study, reducing the neuroprotective 500 μg/kg dose to 170 μg/kg abolished the protective effect of ExAc on the brain. This is in line with our previous study in mice, where reducing the dose by 10-fold did not provide neuroprotection [24]. Similarly, a study by Teramoto and colleagues, using exendin-4 in adult mice to prevent transient focal ischemia, showed a lack of exendin-4-mediated protection when administering a dose below 10 μg/100 μL/mouse, which is equivalent to our dose of 500 μg/kg [28].

Rabbit kits, unlike mice, only nurse once daily, briefly, at nighttime [29]. Therefore, and due to the well-established satiating effect of exenatide, we decided to try both single and dual dose regimens, with a 24 h interval to reduce the risk of underfeeding during the treatment period. Encouragingly, and differently from the rodent studies, we were able to achieve substantial neuroprotection with a 90% reduction in tissue loss with a single exenatide dose. An additional dose 24 h later maintained the same level of protection, reaching 73% tissue loss reduction, confirming again a lack of toxicity associated with this high dose.

Our results also showed that in some litters, specifically where hypoxia-ischemia was performed in P3 rabbit kits, there was a 16.6% mortality within the last 30 min of hypoxia. Therefore, the exact same high-dose ExAc experiment was repeated at P4. Similarly to what was observed with the P3 hypoxia-ischemia, 500 μg/kg exenatide treatment, both as a single dose or as a 2-dose 24 h apart, resulted in substantial neuroprotection, with an average 90% reduction in tissue loss. The induction of hypoxia-ischemia at P4 did not result in any mortality.

Physiological measurements following a single high dose of exenatide showed that, in line with clinical administration, exenatide did not alter glucose levels, which remained homeostatic [30]. Interestingly, blood samples showed that ketone bodies levels were raised transiently at 6 h after ExAc treatment. However, looking at the average increase from 0.6 to 1.3 mmol/L, this represents only mild transient ketonemia, as this effect did not persist when blood ketone bodies were measured at 24 h after administration of ExAc. Clinically, GLP-1R agonists, including exenatide, are known to cause a satiating effect with subsequent weight loss [31]. In our study, a single high dose of ExAc treatment, following hypoxia-ischemia, did not alter body weight when compared to vehicle controls. Additionally, the exenatide treatment did not affect core temperature.

There has been a significant reduction in hypoxia-ischemia incidence in high-income countries, from 1–5/1000 live births in the early 2000s to 0.5–1.9/1000 by 2010, with a recent study indicating a stable incidence in 2019 of 1.7/1000 live births [1]. This improvement is mainly attributed to improvements in both antenatal and postnatal care, including the introduction of therapeutic hypothermia as standard care. However, the incidence of hypoxia-ischemia in low- to middle-income countries has not shown the same trend in reduction. In fact, it has remained high, with 1.5 to 20.3/1000 live births, with a higher rate of severe cases, and higher mortality [1]. Furthermore, therapeutic hypothermia can be difficult to properly implement in these studies, showing no protection or even harmful effects [32,33]. Therefore, the current study was designed to add further evidence, for the potential use of ExAc as a therapeutic agent against hypoxia-ischemia brain injury, that could be of relevance for middle- and low-resource countries where therapeutic hypothermia is not used, and the vast majority of cases occur.

In summary, the aim of the current study was to create a new complementary and more clinically relevant animal model of hypoxia-ischemia, in which we could generate further data to support the use of exenatide as a therapeutic agent against hypoxia-ischemia. Our results are promising and show that ExAc (500 μg/kg) remains neuroprotective across different species, and that the beneficial effect of ExAc is lost if the dose is reduced to 170 μg/kg.

## Data Availability

The data sets will be available upon reasonable request.

## Abbreviations

The following abbreviations are used in this manuscript:

DAB	3.3-di-aminobenzidine
ExAc	Exenatide acetate
GFAP	Glial fibrillary acidic protein
GLP-1R	Glucagon-like peptide-1 receptor
GMP	Good manufacturing practice
IBA1	Ionized calcium-binding adapter molecule 1
I.P.	Intraperitoneal
MAP-2	Mouse anti-microtubule-associated protein 2
NeuN	Neuronal specific nuclear protein
Olig2	Oligodendrocyte transcription factor 2
P	Postnatal day
RUO	Research use only
